# MMP-7蛋白在肺癌患者和正常人外周血中的表达水平及临床意义

**DOI:** 10.3779/j.issn.1009-3419.2012.12.06

**Published:** 2012-12-20

**Authors:** 小亮 孙, 汀 肖, 磊 杨, 燕宁 高, 贵余 程, 克林 孙

**Affiliations:** 1 100021 北京，北京协和医学院，中国医学科学院，肿瘤医院胸外科 Department of Thoracic Surgery; 2 肿瘤研究所“癌发生及预防分子机理”，北京市重点实验室 Beijing Key Laboratory for Carcinogenesis and Cancer Prevention, Cancer Institute (Hospital), Peking Union Medical College and Chinese Academy of Medical Sciences, Beijing 100021, China

**Keywords:** 基质金属蛋白酶7, 肺肿瘤, 血浆, 肿瘤标志物, Matrix metalloproteinase 7 (MMP-7), Lung neoplasms, Plasma, Tumor marker

## Abstract

**背景与目的:**

基质金属蛋白酶7（matrix metalloproteinase 7, MMP-7）又称基质溶解素，是MMPs家族成员之一，本研究旨在检测MMP-7在肺癌患者和正常人外周血血浆中的蛋白水平，并探讨其临床意义。

**方法:**

采用酶联接免疫吸附试验（enzyme-linked immunosorbnent assay, ELISA）检测114例肺癌患者和100名正常人外周血血浆标本中的MMP-7浓度。

**结果:**

肺癌患者外周血血浆中的MMP-7蛋白浓度（*n*=114, median=0.72 ng/mL）明显高于正常人外周血血浆中的MMP-7蛋白浓度（*n*=100, median=0.30 ng/mL, *P* < 0.001），当cutoff值为0.56 ng/mL时，MMP-7检测肺癌的敏感性为62.3%，特异性为76.0%。但是，肺癌患者外周血血浆中MMP-7的蛋白水平与患者的年龄、性别、吸烟史、肿瘤大小、病理类型、淋巴结转移及分期均无关（*P* > 0.05）。

**结论:**

外周血血浆中MMP-7可以作为辅助肺癌诊断的一种肿瘤标志物，但其与肺癌的各项临床参数之间无明显联系，需要进一步扩大样本进行分析。

## Abstract (en)

**Background and objective:**

Matrix metalloproteinase 7 (MMP-7), also known as matrilysin, is a member of the MMP family. The objectives of this study were to test MMP-7 protein levels in the peripheral blood of lung cancer patients and healthy control subjects and to determine their corresponding clinical significance.

**Methods:**

Peripheral blood samples were obtained from 114 lung cancer patients and 100 healthy control subjects. MMP-7 protein levels in the plasma were measured by enzyme-linked immunosorbent assay.

**Results:**

The plasma protein levels of MMP-7 in lung cancer patients (median=0.72 ng/mL) were significantly higher than those in healthy control subjects (median=0.30 ng/mL)(*P* < 0.001). When the cutoff of MMP-7 protein level was set at 0.56 ng/mL, the sensitivity and specificity of detecting lung cancer were 62.3% and 76.0%, respectively. However, the lung cancer patients and healthy control subjects did not statistically differ in age, sex, smoking status, tumor size, pathological classification, as well as lymphatic metastasis and stage (*P* > 0.05).

**Conclusion:**

The plasma protein levels of MMP-7 increase in the peripheral blood of lung cancer patients. Peripheral blood MMP-7 can be used as a tumor marker for detecting lung cancer. The fact that no significant correlation between the protein levels of MMP-7 and lung cancer clinical parameters was observed in this study warrants further analysis in larger samples.

最新统计数据^[1]^显示，肺癌已成为美国男性和女性发病率第二位的恶性肿瘤，死亡率居各恶性肿瘤之首，成为严重危害人类健康的疾病之一。非小细胞肺癌（non-small cell lung cancer, NSCLC）是肺癌最常见的类型，占肺癌的85%，但5年生存率却仅为15%。70%的肺癌患者在确诊时已属晚期，失去了手术机会^[2]^。如果肺癌患者能早期诊断，5年生存率可达80%^[3]^。基质金属蛋白酶-7（matrix metalloproteinase 7, MMP-7）又称基质溶解素，是MMPs家族成员之一，具有一定的底物特异性，主要作用于细胞外基质、基底膜及细胞膜表面分子等，它可在人体正常上皮细胞中表达，但活性非常低。MMP-7的过表达发生于各种上皮来源的肿瘤及间质肿瘤，其过表达与肿瘤细胞的发生、浸润、转移和复发等密切相关^[4]^。

目前，肺癌组织中MMP-7蛋白表达情况的检测国内外有较多的报道，血清中MMP-7的RNA检测近来也有少量报道，但尚未见关于外周血血浆中MMP-7的蛋白水平的研究，本研究旨在通过双抗体ELISA夹心法检测肺癌患者和正常人外周血血浆中MMP-7的蛋白水平，以探讨其是否可以作为肺癌的肿瘤标志物及其与肺癌的关系。

## 1 材料与方法

### 1.1 临床资料

实验组为2010年3月-2011年5月在中国医学科学院肿瘤医院确诊的肺癌患者114例。入组标准：①经支气管镜活检、细胞学或术后病理证实为肺癌患者；②未接受过放化疗治疗；③无严重肺间质性疾病。根据世界卫生组织（World Health Organization, WHO）2004年的肺癌组织学分型标准进行肺癌组织学分型，根据国际抗癌联盟（International Union Against Cancer, UICC）2009年发布的第7版肺癌TNM分期系统进行肺癌分期。114例肺癌患者包括男性77例，女性37例；中位年龄60岁（38岁-78岁）；既往有吸烟史65例，无吸烟史49例；肿瘤直径≤3 cm 53例， > 3 cm 61例；鳞癌42例，腺癌52例，小细胞肺癌10例，其它（类癌、支气管内粘液表皮样癌、非典型类癌）3例，无明确病理类型7例；低分化38例，中分化47例，高分化8例，无明确分化21例；无淋巴结转移40例，有淋巴结转移59例，淋巴结转移不详15例；Ⅰ期+Ⅱ期61例，Ⅲ期+Ⅳ期53例。详细临床资料见表 1。

**1:** 肺癌患者外周血中MMP-7蛋白浓度 The results of MMP-7 concentration in lung cancer patients

Characteristic	*n*	Median	*Z*	*P*
Age (years)				
≤60	55	0.76	-0.045	0.964
> 60	59	0.70		
Gender				
Male	77	0.72	-0.369	0.712
Female	37	0.71		
Smoking history				
Yes	65	0.73	-0.458	0.641
No	49	0.68		
Size of tumor				
≤3 cm	53	0.62	-1.116	0.264
> 3 cm	61	0.83		
Pathological type^a^				
SCC	42	0.71	-0.654	0.513
ADC	52	0.88		
Grade^b^				
High+Moderate	55	0.68	-1.043	0.297
Poor	38	0.87		
Lymph node metastasis^c^				
Yes	59	0.76	-0.053	0.957
No	40	0.70		
Stage				
Ⅰ+Ⅱ	61	0.70	-0.676	0.499
Ⅲ+Ⅳ	53	0.87		
^a^There are some other pathological type, such as SCLC, carcinoid tumor, etc (20). The number of these types is limited, so they were not included in this table. ^b^There are some patients (21) whose tumor grade is not clear, so they were not included in this table. ^c^There are some patients (15) whose lymph node metastasis is not clear, so they were not included in this table. SCLC, small cell lung cancer; ADC, adenocarcinoma.				

对照组为中国医学科学院肿瘤医院防癌科体检的100例性别和年龄匹配的正常人群，既往均无严重肺间质疾病。

### 1.2 ELISA方法

用EDTA抗凝管采肺癌患者空腹静脉血，置4 ℃冰箱暂存，2 h内送实验室，经低温离心（1, 500 rpm, 4 ℃, 10 min）分离，取上层血浆分装后保存-80 ℃冰箱待检。对照组正常人的外周血样品由中国医学科学院肿瘤医院防癌科提供。应用ELISA双抗体夹心法检测外周血血浆中MMP-7蛋白水平（按照MMP-7 ELISA KIT[美国R&D公司，货号为SMP700]说明书进行）。操作步骤如下：从室温平衡20 min后的铝箔袋中取出所需板条，剩余板条用自封袋密封放回4 ℃冰箱；标准蛋白浓度：标准品（S0-S8）浓度依次为0 ng/mL、0.156 ng/mL、0.312 ng/mL、0.625 ng/mL、1.25 ng/mL、2.5 ng/mL、5 ng/mL、10 ng/mL。样本孔先加样本稀释液25 μL，再加待测样本25 μL，标准品孔加50 μL标准品，空白孔加PBS液，置于水平振荡器（500±50）rpm室温孵育2 h。洗板去除多余样品，1×PBST缓冲液洗板4次。加酶标抗体：每个反应孔加200 μL，置于水平振荡器室温孵育2 h。洗板去除多余抗体，1×PBST缓冲液洗板4次。显色：每个反应孔中分别加入200 μL新鲜配置的显色液（A+B），室温避光反应30 min。终止反应：每个反应孔加入终止液50 μL，终止反应。在酶标仪上于450 nm处测定各孔的OD值。标准曲线的建立：分别以浓度为0、0.156 ng/mL、0.312 ng/mL、0.625 ng/mL、1.25 ng/mL、2.5 ng/mL、5 ng/mL和10 ng/mL的MMP-7标准蛋白为S0-S8标准点，检测其OD值，以S0点为空白对照，以S0-S8标准品的浓度为横坐标，相应的OD值为纵坐标，制作出标准品线性回归标准曲线，求得标准曲线公式及相关系数，根据公式计算相应标准品的浓度值（ng/mL）。

### 1.3 统计学方法

采用SPSS 17.0统计软件进行统计学分析。计量资料进行正态性检验，不符合正态分布的数据进行非参数检验（*Mann-Whitney U test*），符合正态分布的数据进行t检验。*P* < 0.05为差异有统计学意义。

## 2 结果

### 2.1 肺癌患者与正常人外周血血浆中MMP-7的蛋白水平

肺癌患者外周血血浆中MMP-7蛋白浓度（*n*=114, median=0.72 ng/mL）明显高于正常人外周血血浆中MMP-7蛋白浓度（*n*=100, median=0.30 ng/mL），两组之间差异有统计学意义（*Z*=-6.649，*P* < 0.001，图 1）。

**1:**
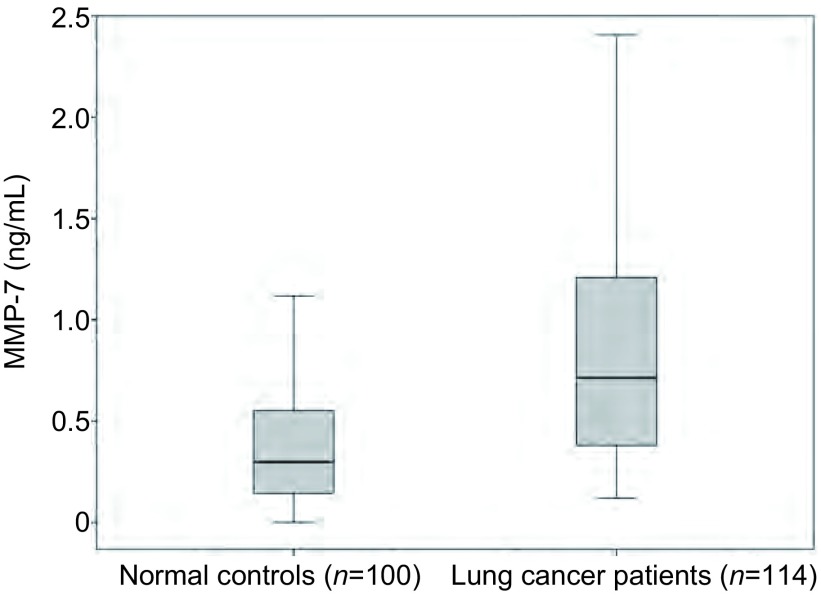
肺癌患者和正常人外周血中MMP-7蛋白浓度 MMP-7 concentrations in lung cancer patients and normal controls

### 2.2 肺癌患者外周血血浆中MMP-7的蛋白浓度与肺癌患者临床特征的关系

将肺癌患者按照临床特征分组，分别比较各组间MMP-7蛋白浓度的差异，结果（表 1）提示：年龄（≤60岁和 > 60岁）、性别（男和女）、吸烟史（有和无）、肿瘤大小（≤3 cm和 > 3 cm）、病理类型（鳞癌、腺癌、小细胞肺癌等）、淋巴结转移（有和无）及分期（Ⅰ期+Ⅱ期和Ⅲ期+Ⅳ期）各组的肺癌患者外周血血浆中MMP-7蛋白浓度差异无统计学意义（*P* > 0.05）。

### 2.3 以血浆中MMP-7浓度作为诊断标准的诊断效能

采用血浆中MMP-7浓度区分肺癌病例和正常人群时，接受者操作特性曲线（receiver operating characteristic curve, ROC）的曲线下面积（area under the ROC curve, AUC）为0.764，当cutoff值定为0.56 ng/mL，MMP-7检测肺癌的敏感性为62.3%，特异性为76.0%（图 2）。

**2:**
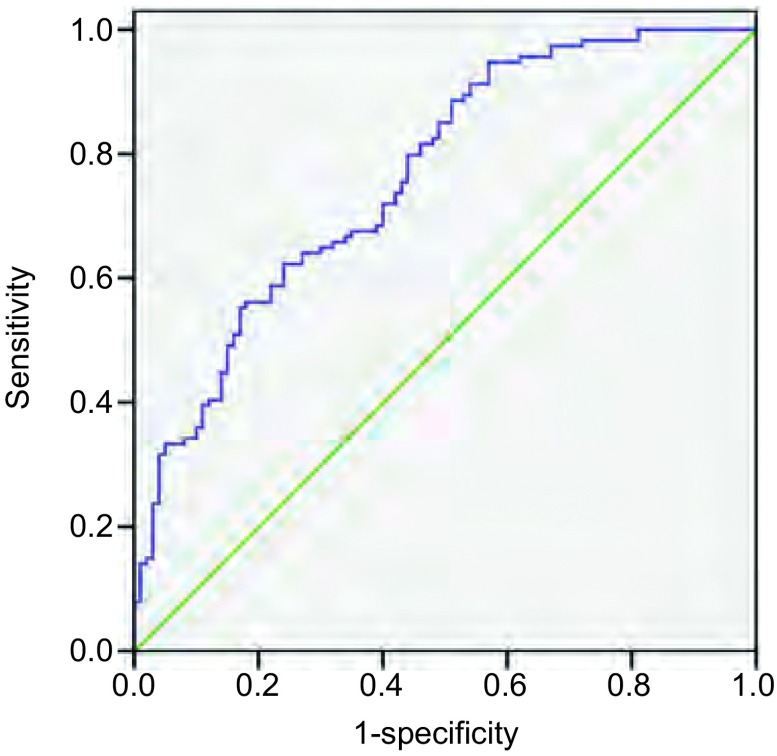
采用血浆中MMP-7浓度区分肺癌患者和正常人的ROC曲线 The ROC curve of MMP-7. ROC: receiver operating characteristic curve.

## 3 讨论

进入21世纪以后，肺癌已经超过胃癌和肝癌成为我国恶性肿瘤死因的第一位^[5]^。2005年我国肺癌新发病例数为536, 407人，死亡病例数475, 768人，年平均增长1.63%^[6]^，早期肺癌经积极治疗，5年生存率可达60%以上，但肺癌的总体5年生存率却低于15%，主要原因是肺癌发病隐匿，多数在诊断时已属晚期。因此，研究肺癌的生物学特性对肺癌的诊治显得尤为重要。

基质金属蛋白酶（MMPs）是一类具有Zn^2+^依赖性的内源性蛋白水解酶，几乎能降解除多糖外细胞外基质的所有成分。自1962年Gross等人首先发现第一种间质胶原酶并命名为MMP-1后，至今在MMP家族中至少已发现25个成员^[7]^。MMP-7是一种分泌型蛋白，由Sellers和Woessner于1980年在大鼠子宫中发现，1988年第一次成功克隆出人的同源基因^[8]^。人类MMP-7基因定位于11q21-q22，与MMPs家族其它MMP相比较，MMP-7分子量只有28 kDa，是目前所发现的相对分子质量最小的MMPs。MMP-7在消化道肿瘤的进展期存在过表达现象，如食管^[9]^、胃^[10]^、结肠^[11]^、肝^[12]^和胰腺^[13]^。Liu等^[14]^研究了MMP-7在人类肺腺癌A549细胞系中的表达及作用，提示MMP-7与肺癌细胞增殖、凋亡相关。

本研究通过ELISA法检测114例肺癌患者和100例正常人外周血血浆中MMP-7蛋白水平，证实肺癌患者外周血血浆中MMP-7蛋白水平明显高于正常人外周血血浆中MMP-7蛋白水平（*P* < 0.001），当cutoff值为0.56 ng/mL时，MMP-7检测肺癌的敏感性为62.3%，特异性为76.0%，说明MMP-7可能成为一种辅助肺癌诊断的肿瘤标志物，并有希望成为一种新的肺癌治疗的靶向药物的靶标。但因MMP-7表达于各种上皮来源的肿瘤及间质肿瘤，无特异性，故目前尚无证据表明其可以早期诊断肺癌。况且，肺癌的早期诊断是一项重大的挑战，尤其是单一标志物更难以做到。

目前，已有多项研究^[15-17]^报告证实肺癌组织中MMP-7的表达明显高于正常肺组织，且肺癌组织中MMP-7的表达与肿瘤的分化、淋巴结转移、分期及预后等密切相关，但也有研究^[18]^表明肺癌组织中MMP-7的表达与肿瘤的淋巴结转移及分期等无关。但尚未见有肺癌患者外周血中MMP-7蛋白水平与肿瘤分化、淋巴结转移及分期等生物学特性的相关文章报道。本研究通过ELISA法检测肺癌患者外周血中MMP-7蛋白浓度，然后根据肺癌的生物学特性进行分组分析，结果表明肺癌患者外周血中MMP-7蛋白浓度与肺癌患者的年龄、性别、有无吸烟史、肿瘤大小、病理类型、淋巴结有无转移及分期均无明显相关性，分析原因可能为：①样本量的限制；②目前尚无证据证明肺癌组织中MMP-7表达与外周血中MMP-7蛋白水平有一致性；③外周血中的肿瘤标志物仅具有辅助诊断的作用，与肿瘤的生物学特性本来就没有直接的关系（目前已被临床广泛接受并应用的外周血PSA、AFP等标志物可以辅助甚至早期诊断前列腺癌、肝细胞肝癌等，但临床已证实其浓度与肿瘤的生物学特性无相关性）。因此，尚需要更进一步大样本的临床研究证实。
